# Removal of Malachite Green by Poly(acrylamide-co-acrylic acid) Hydrogels: Analysis of Coulombic and Hydrogen Bond Donor–Acceptor Interactions

**DOI:** 10.3390/gels9120946

**Published:** 2023-12-01

**Authors:** Salah Hamri, Bouchra Bouzi, Djahida Lerari, Fayçal Dergal, Tewfik Bouchaour, Khaldoun Bachari, Zohra Bouberka, Ulrich Maschke

**Affiliations:** 1 Center for Scientific and Technical Research in Physico-Chemical Analysis (CRAPC), BP 384, Industrial Zone, 42004 BouIsmaïl, Algeria; 2 Macromolecular Research Laboratory (LRM), Faculty of Sciences, Abou Bekr Belkaid University, BP 119, 13000 Tlemcen, Algeria; 3 Laboratoire Physico-Chimie des Matériaux-Catalyse et Environnement (LPCMCE), Université des Sciences et de la Technologie d’Oran Mohamed Boudiaf (USTOMB), BP 1505, 31000 Oran, Algeria; 4 Unité Matériaux et Transformations—UMET, UMR 8207, Université de Lille, CNRS, INRAE, Centrale Lille, 59000 Lille, France

**Keywords:** wastewater, contaminant, copolymers, swelling, docking

## Abstract

Water pollution caused by dyes poses a significant threat to life on earth. Poly(acrylamide-co-acrylic acid) hydrogels are widely used to treat wastewater from various pollutants. This study aims to examine the removal of malachite green (MG), a harmful and persistent dye that could cause extensive environmental damage, from an aqueous solution by adjusting the initial concentration of acrylamide (AM) and the degree of copolymer crosslinking. The copolymer hydrogels efficiently eliminate MG in a brief timeframe. The most successful hydrogel accomplished a removal rate exceeding 96%. The copolymer of 4 wt % 1,6-hexanediol diacrylate and a concentration of 100 mg/mL AM was effective. The degree of swelling was affected by crosslinking density as expected, with low crosslinking ratios resulting in significant swelling and high ratios resulting in less swelling. To evaluate the results, a docking approach was used which presented three crosslinked models: low, medium, and high. The copolymer–dye hydrogel system displayed robust hydrogen bonding interactions, as confirmed by the high quantities of both donors and acceptors. It was determined that MG contains six rotatable bonds, enabling it to adapt and interact with the copolymer chains. The dye and copolymer enhance H-bond formation by providing two hydrogen bond donors and 16 hydrogen bond acceptors, respectively. Through capitalizing on cationic and anionic effects, the ionic MG/copolymer hydrogel system improves retention efficiency by enhancing attraction between opposing charges. It is interesting to note that the synthesized copolymer is able to remove 96.4% of MG from aqueous media within one hour of contact time.

## 1. Introduction

Water is the main component of the earth’s hydrosphere, utilized by humans for consumption, cleaning, and cooking, as well as by animals and plants to sustain life [1,2,3]. The oxygen atom in the water molecule maintains a negative charge, while the hydrogen atoms have a positive charge, causing the molecule to have an electric dipole moment and be classified as a polar molecule [4]. Water serves as a solvent for various salts and hydrophilic organic molecules, including dyes. The presence of dyes in waterways significantly impacts living organisms. Dyes are extensively utilized across industries at various scales, and untreated wastewater effluents frequently discharge, leading to river and water source contamination [5,6,7,8].

To address this pollution, techniques including oxidation, flocculation, coagulation, and absorption have been implemented to remove dyes from water [9,10,11,12,13]. However, absorption remains a relatively inexpensive method for removing dyes from water, in contrast to more costly alternatives. Typically, materials used for absorption exhibit a hydrophilic nature that allows them to absorb at least 20% of water without dissolution [14].

Several materials have been utilized to eliminate various waterborne contaminants like dyes, chemicals, trash, bacteria, and parasites. Ali et al. [15] effectively used a selectively designed carboxymethylcellulose macromolecule iron composite to extract atorvastatin drug residues from water medium, leading to an 80% removal rate. Similarly, in their research [16], multi-walled carbon nanotubes were employed to achieve a 90% removal of the funoron pesticide from water. Azam et al. [17] utilized the Adjwa date to extract Cu(II) ions from an aqueous solution, resulting in the removal of 80–89% of the ions. Meanwhile, Khan et al. [18] developed a nano-magnetic composite of copper ferrite/drumstick pod biomass (CuFe_2_O_4_/DC) using the co-precipitation procedure. This composite was optimized and characterized to effectively sequester malachite green (MG) and lead (Pb(II)) in both unary and binary systems from aqueous media. Pb(II) adsorption decreased by 82.7% after the initial regeneration cycle, whereas MG adsorption only decreased by 17.6% after five consecutive regeneration cycles. In a previous study [19], cryogenic silico-manganese (Si-Mn) fume beads (cSMFB) were developed from SMF waste precursors using a sustainable and cost-effective method. The resulting material was then employed for the removal of cationic dyes (methylene blue (MB), methylene green (GM), crystal violet (CV), and celestine blue (CB) from an aqueous environment in a batch operating mode. Results indicated that the adsorption of dyes may be influenced by electrostatic interaction, coordinate bond formation, and cationic interchange.

Numerous dyes used in diverse applications have been subjected to hydrogel composites and nanocomposites [20]. The polyacrylamide (AM)-co-acrylic acid (AA) copolymer was effectively used to eliminate Safranine-O SO and Magenta M dyes [21]. In a previous study [22,23], a crosslinked poly(AM-co-AA)/1,6-hexanediol diacrylate (HDDA) hydrogel was applied to remove Rose Bengal (RB), MB, and Eosin Y (EY), resulting in retention percentages of 98%, 50%, and 58%, respectively. The method employed is effective and promising in eliminating these dyes. MG is a bright green dye commonly utilized in treating fungal infections in fish, particularly in aquaculture and the ornamental fish sector. MG has shown a tendency to endure in the environment, particularly in water, soil, and sediment, and has been detected in groundwater, rivers, and lakes. As a consequence, MG can accumulate within the environment, resulting in extended exposure for both wildlife and human populations. Additionally, certain organizations, such as the International Agency for Research on Cancer (IARC) [24,25], have designated MG as a potential carcinogen to humans. Consequently, the use of MG in veterinary medicine has been restricted in several countries, and attempts are underway to uncover safer and more ecologically friendly dyes. Best practices must be implemented throughout the manufacturing, usage, and disposal phases of MG to minimize its impact on the environment. Proper treatment and disposal of industrial effluents containing MG can significantly reduce its harmful effects [26,27].

The removal of MG with composite hydrogels has been the subject of research. For instance, carboxymethyl cellulose-based graft poly(AM) hydrogel (CMC-g-P(AM)) and its nanocomposite with montmorillonite (CMC-g-P(AM)/MMT) demonstrate effectiveness [28]. Furthermore, hydrogel materials made from chitosan and carboxymethyl cellulose were enhanced with bentonite. The hydrogel materials synthesized in the study underwent an evaluation for their effectiveness in removing toxic dyes, such as MG and RB. The adsorption process yielded a 96% removal rate for MG [29]. Layth et al. [30] synthesized a hydrogel using poly(AM-maleic acid) through radical polymerization of AM and maleic acid monomers. N,N-methylenebisacrylamide served as a crosslinker and potassium persulfate as an initiator. Samples were prepared through thermal polymerization at a temperature of 60 °C. The resulting adsorbents demonstrated high efficacy in removing MG, with an exceptional adsorption capacity. The adsorption mechanism followed pseudo-second-order kinetics [30]. Additionally, numerous studies have employed the second-order Lagrange model to investigate the diffusion behavior of dye within the hydrogels and to gather vital data about key parameters, including the diffusion equation, the diffusion coefficient D, and others [31,32,33,34,35].

Copolymer hydrogels have been utilized in numerous research studies for the removal of MG dye. Machado et al. synthesized copolymers via thermal polymerization and crosslinked them with bisacrylamide [36]. It has been demonstrated that the material properties are greatly influenced by the choice of polymerization method [37]. Photopolymerization technologies can replace many traditional techniques that use heat curing and solvents. The benefits of photopolymerization outweigh those of thermosetting polymerization due to faster polymerization rates and a safer environment, contributing to the elimination of volatile organic solvents [38]. Notably, UV curing has exponentially increased in industrial processes in recent decades.

When studying poly(AM-co-AA), researchers have typically varied the amount of AA while maintaining a constant initial AM concentration [39]. Varying the initial concentration of AM can impact material porosity. This study produced photochemically crosslinked copolymers of poly(AM-co-AA)/HDDA through UV irradiation of the corresponding monomers. To examine MG retention in the copolymer hydrogels, both the initial AM concentration and crosslinking degree were varied. Different levels of HDDA were added to the initial monomer blends to vary the latter.

A simulation was conducted to examine the swelling properties and retention of MG by copolymer hydrogels. Three models were studied, with variations in degree of crosslinking being taken into consideration. Three parameters, namely, the number of rotatable bonds, hydrogen bond donors (HBDs), and hydrogen bond acceptors (HBAs), were examined for the first time to explain the dye retention.

The number of rotatable bonds in a molecule is a crucial physicochemical property that impacts its effectiveness and capacity for adsorption or liberation. Rotatable bonds refer to single bonds that have the ability to freely rotate around their axes, thereby determining the molecule’s conformational flexibility.

The number of rotatable bonds in the MG molecule can affect its interaction with poly(AM-co-AA) and its adsorption properties. While a high number of rotatable bonds in molecules may result in excessive flexibility, leading to poor binding to their target, a low number of such bonds may lead to rigidity and hinder their ability to adapt to the binding site. The study showed that the MG and copolymer, with 6 and 121 rotatable bonds, respectively, demonstrated the necessary flexibility for improving the adsorption process of the dye and receptor.

The number of donor–acceptor hydrogen bond sites is another factor that may account for retention. An acceptor atom possesses one or more lone pairs of electrons that can participate in H-bonding, whereas the donor “donates” its hydrogen to the acceptor. In this system, the MG includes two HBDs, while the copolymer model has 16 HBAs. This further promotes hydrogen bond formation in the copolymer/dye system.

## 2. Results and Discussion

### 2.1. MG Absorbance on Crosslinked Poly(AM-co-AA)

**Figure 1:**
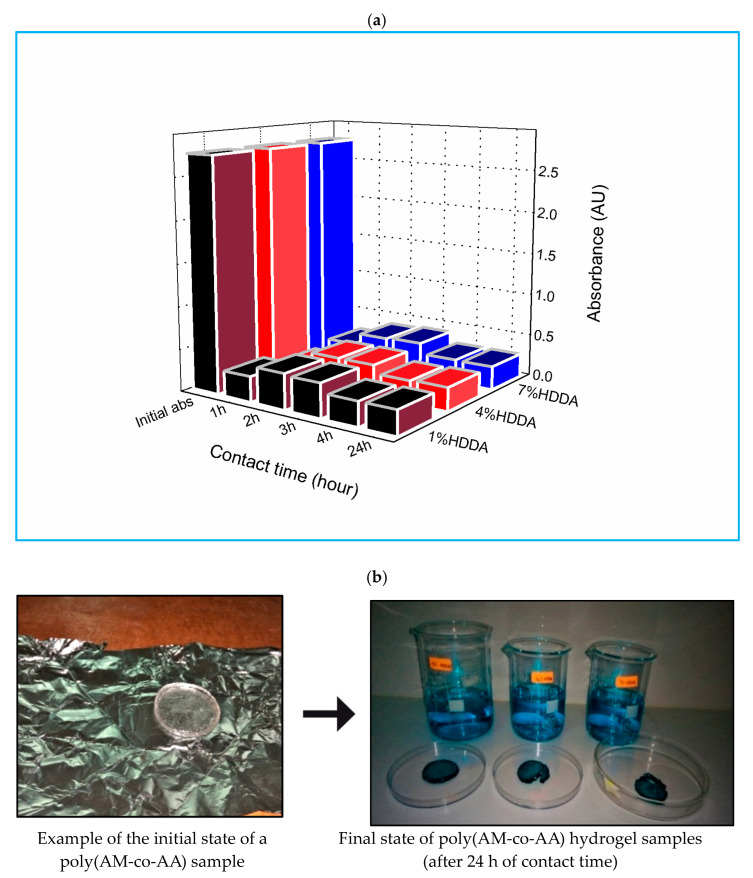
(**a**) Evolution of MG absorbance in the presence of poly(AM_C1_-co-AA) hydrogel as a function of crosslinker content and contact time. (**b**) The pellets have turned green in color after being in contact for 24 h.

Figure 1a illustrates the changes in MG absorbance over time when in contact with poly(AM_C1_-co-AA) hydrogel. Additionally, data from Figures S1 and S2 and Table S1 were referenced in this study. The initial absorbance for all samples was 2.76. The absorbance values were measured for different concentrations of HDDA copolymer, specifically at 1, 4, and 7 wt %, after a 1 h contact time. The copolymer hydrogel, which was prepared using 1 wt % HDDA, displayed an absorbance of 0.30, whereas the absorbance was 0.19 and 0.25 for hydrogels prepared with 4 wt % and 7 wt % HDDA, respectively. Among all the samples, the 4 wt % HDDA hydrogel exhibited the lowest absorbance after 1 h of contact time (0.28), and this value remained the lowest, even after 24 h, as outlined in Table S2. The ideal immersion duration for MG is 1 h since the absorbance rises after 1 h.

**Figure 2:**
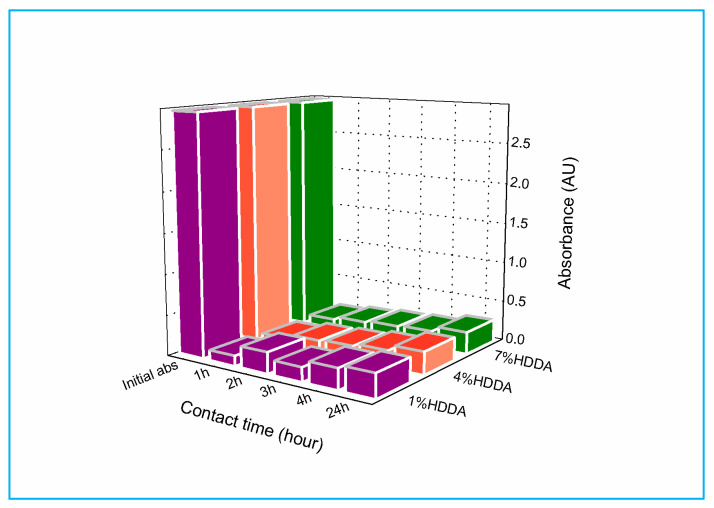
Evolution of MG absorbance in the presence of poly(AM_C2_-co-AA) hydrogel as a function of crosslinker content and contact time.

Figure 2 demonstrates the impact of the initial HDDA concentration in poly(AM_C2_-co-AA) on the absorption of MG over a 24 h period. In the beginning, the absorbance of the dye solutions decreased. However, after the 2 h mark, it began to rise, resulting in absorption rates of 0.25 (1 wt % HDDA), 0.15 (4 wt % HDDA), and 0.16 (7 wt % HDDA). The absorbance of the dye solutions continued to slowly increase until the 24 h mark, resulting in absorption rates of 0.29 (1 wt % HDDA), 0.28 (4 wt % HDDA), and 0.27 (7 wt % HDDA) (refer to Table S3). The hydrogel containing 4 wt % HDDA displayed the highest rate of MG removal, exhibiting an absorbance of 0.09 after a contact duration of 1 h, indicating that high MG removal is characterized by low absorbance.

**Figure 3:**
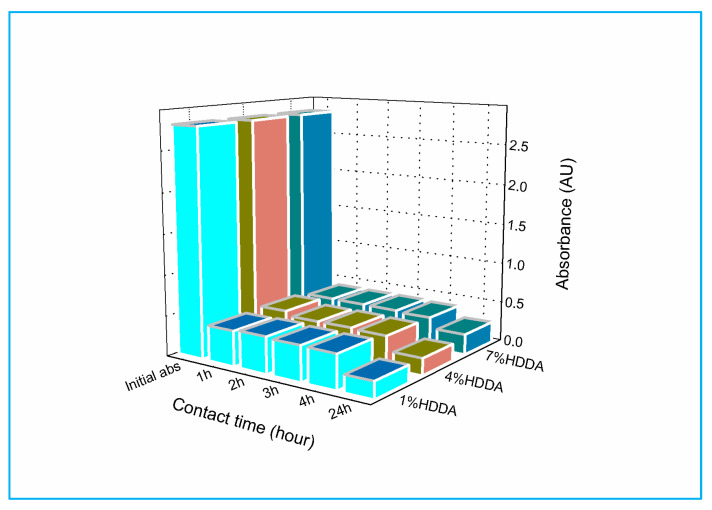
Evolution of MG absorbance in the presence of P(AM_C3_-co-AA) hydrogel as a function of crosslinker content and contact time.

Figure 3 illustrates a reduction in dye solution absorbance within the three hydrogels. Precisely at 1 h contact time, the 1 wt % HDDA hydrogel displayed marginally higher absorbance (0.44) than the 4 wt % and 7 wt % HDDA hydrogels (0.40). The absorbance of the solutions decreased continuously to 0.20 for the 1 wt % and 4 wt % HDDA samples, and to 0.25 for the 7 wt % HDDA sample after 24 h of contact time (refer to Table S4 for further information). For this specific scenario, an optimal contact time of 24 h is recommended, and the hydrogel containing 1 wt % of HDDA is the most efficient for MG removal.

**Figure 4:**
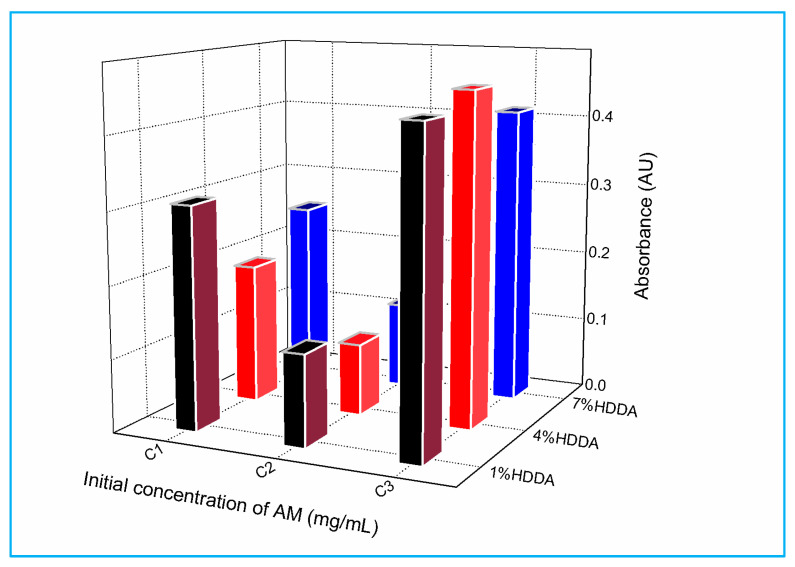
Effect of AM and HDDA concentrations on the absorbance of MG in the presence of poly(AM-co-AA) hydrogel, after 1 h of contact time.

Figure 4 exhibits the absorbance of MG in the presence of the poly(AM-co-AA) hydrogel after one hour of contact time as a function of AM and HDDA concentrations. All the copolymer compositions displayed high removal rates of MG, exceeding 83%. The hydrogel copolymer with a C2 concentration of AM, as shown in Table 1, achieves the greatest removal of MG at 96.4%, 95.7%, and 95.5% for the copolymer with 4, 7, and 1 wt % of HDDA, respectively.

In contrast, concentration C1 experiences a decline in dye removal, while concentration C3 displays a relatively low percentage of removal.

### 2.2. Ionic Interaction in the Poly(AM-co-AA)/MG Hydrogel

**Figure 5:**
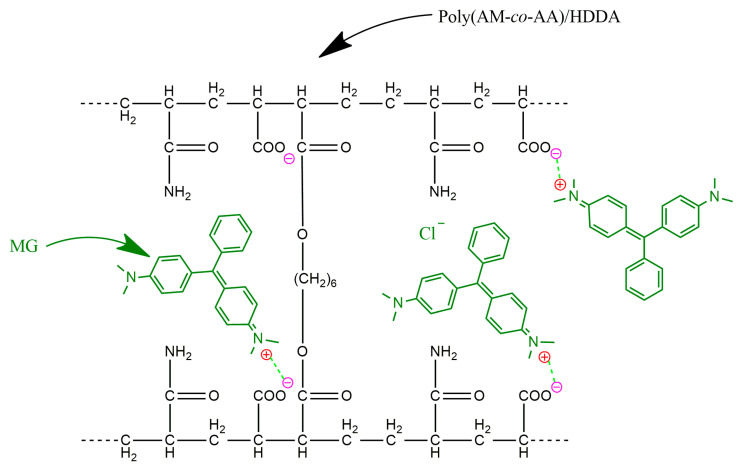
Ionic interaction between MG (in red color: positive charge) and the carboxyl group COO^−^ (in pink color: negative charge).

The electrostatic attraction between oppositely charged groups is known as ion–ion interaction or ionic bonding. These interactions are responsible for maintaining the stability of ionic compounds. Opposingly charged ions attract each other while similarly charged ions repel each other [40]. Figure 5 exemplifies the creation of ionic bonds between positively and negatively charged molecules. The positive charge of the MG is attracted to the negative charge of the copolymer chain, particularly to the AA repeating unit containing the anionic carboxyl group, COO^−^. These coulombic forces persist for a prolonged duration and are proportional to the product of the charges (*q*_1_, *q*_2_) divided by the square of the separation distance (*r*^2^), as outlined in Equation (1).

(1) $$F=k_{e}\frac{q_{1}q_{2}}{r^{2}}\;\;\;\;\;\;\;\;\;\;\;\;\;\;\;\;\;$$

where *k_e_* is the Coulomb constant (*k_e_* ≈ 8.988 × 10^9^ N⋅m^2^⋅C^−2^).

Two particles carrying opposite charges are drawn towards each other, and the force of attraction intensifies as they draw closer until they ultimately form a bond. A considerable amount of energy is required to separate them [41]. This phenomenon explains the high affinity of the ionic cross-linked copolymer for retaining MG.

### 2.3. Effect of Initial Monomer Concentration on Equilibrium Swelling

**Figure 6:**
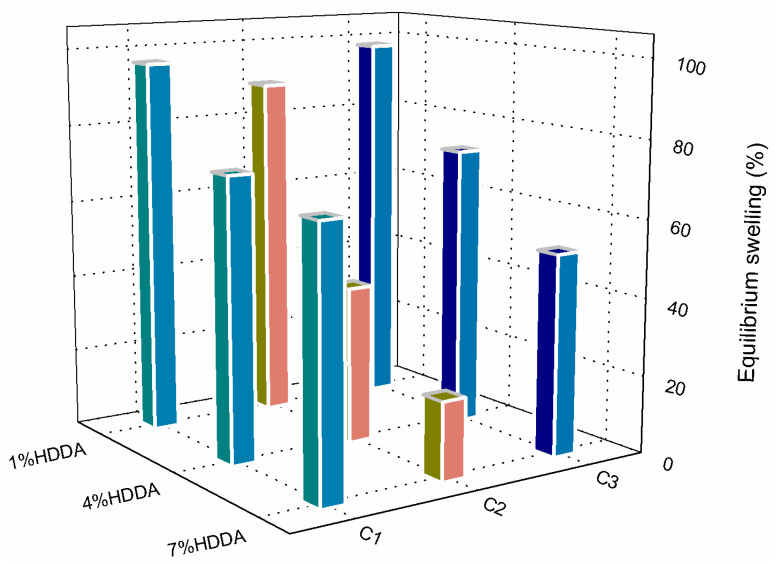
Effect of AM and HDDA concentrations on the equilibrium swelling (ES) of poly(AM-co-AA) hydrogel in MG solution after 24 h contact time.

Figure 6 illustrates the impact of HDDA composition on the equilibrium swelling (ES) of poly(AM-co-AA) hydrogel after 24 h of swelling in dye solutions. The hydrogel with a low crosslink density (1 wt % HDDA) exhibits high swelling for all initial concentrations of AM. Specifically, the swelling percentages for C1, C2, and C3 were 96.5%, 89.1%, and 97.9%, respectively. Meanwhile, increasing the HDDA concentration to 4 wt % results in reduced ES values for all concentrations, with further reductions observed at 7 wt % HDDA (refer to Table 2).

The significant swelling efficiency at C1 concentration, the highest utilized in this study for AM, can be attributed to the corresponding high OH group concentration, attracting water through hydrogen bonding. Thus, there is significant water diffusion within poly(AM_C1_-co-AA) hydrogel.

Figure 6 shows a U-shaped pattern where the minimum is at AM concentration C2, for low, medium, and high crosslinking. These minima exhibit greater MG absorption and lesser water diffusion, which can impact the ES in distilled water via interaction between the two components.

### 2.4. Molecular Docking Analysis

#### 2.4.1. Donor and Acceptor Sites to Build the Hydrogen Bond

Hydrogen bonding is a distinct form of dipole–dipole interaction between molecules, and should not be confused with a covalent bond to a hydrogen atom. This attraction results from the force between a hydrogen atom that is covalently bonded to a highly electronegative atom, such as N, O, or F, and another highly electronegative atom. Hydrogen bond strengths can range from 4 kJ to 50 kJ per mole of hydrogen bonds.

Water is capable of forming strong hydrogen bonds since it can function as both a hydrogen bond acceptor and a donor. This quality contributes to the copolymer hydrogel’s high swelling through water diffusion.

Hydrogen bonds arise from electrostatic attraction between an electronegative atom in one molecule and a proton in another. The hydrogen bond acceptor and donor are the two kinds of compounds involved in hydrogen bond formation.

**Figure 7:**
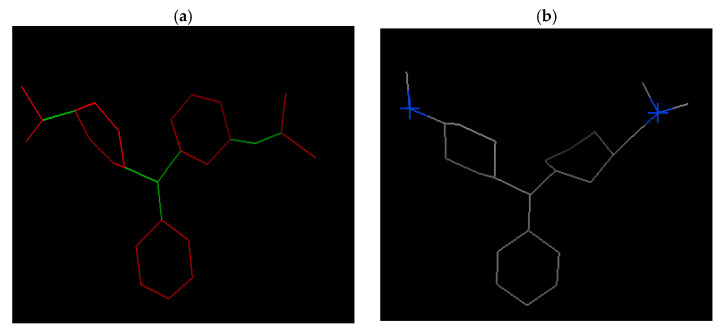
(**a**) Rotatable bonds of MG shown by the docking software. Green: rotatable bond, red: unrotatable bond (6 rotatable bonds). (**b**) Site donor of hydrogen bond: no sp2 donors found, no sp2 acceptors found, no sp3 acceptors found, the plus (+) symbol in blue: sp3 donor found equal to 2, gray: carbon-carbon bond, blue: nitrogen atom.

The docking software identified two sp3 donors for the MG molecule to form a hydrogen bond with the acceptor site on the poly(AM-co-AA)/HDDA copolymer hydrogel. Notably, the software did not find any sp2 donors or sp3 acceptors. MG possesses six rotatable bonds that enable it to interact with the copolymer hydrogel in several conformations (see Figure 7).

**Figure 8:**
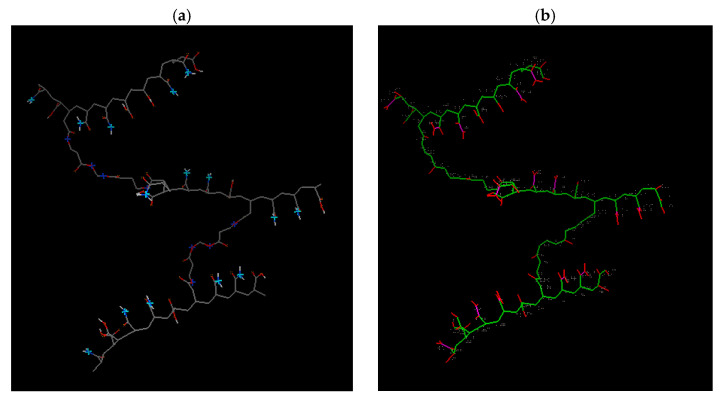
Results from Model 1 (2-HDDA model): (**a**) Donor and acceptor sites for hydrogen bonding (Light blue: acceptor, dark blue: donor, gray: carbon-carbon bond, red: oxygen atom, white: hydrogen atom). (**b**) Rotatable bonds of this model as shown by the docking software (Green: rotatable bond, red: unrotatable bond, magenta: non-rotatable bond).

The diagram in Figure 8 illustrates Model 1, which possesses 16 acceptor sites that form hydrogen bonds and 121 rotatable bonds. These properties aid in the bonding of the dye to the hydrogen. In the experimental section, a robust retention of MG was observed, and the elevated number of acceptor sites rationalizes this result.

**Figure 9:**
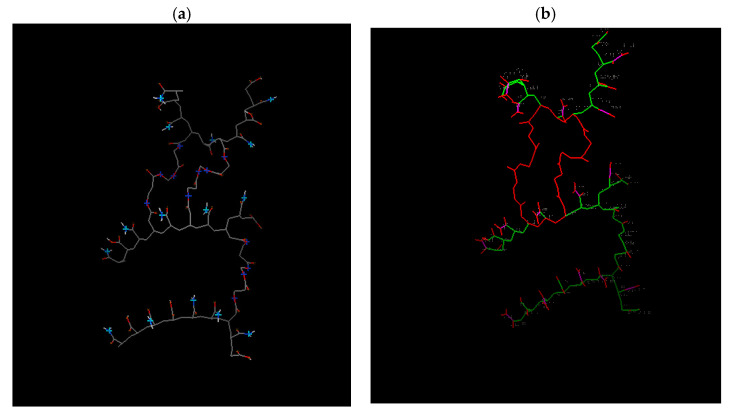
Results from Model 2 (3-HDDA model): (**a**) Hydrogen bonding donor and acceptor sites. (Light blue: acceptor, dark blue: donor, gray: carbon-carbon bond, red: oxygen atom, white: hydrogen atom). (**b**) Rotatable bonds of this model as displayed by the docking software (Green: rotatable bond, red: unrotatable bond, magenta: non-rotatable bond); 91 rotatable bonds.

**Figure 10:**
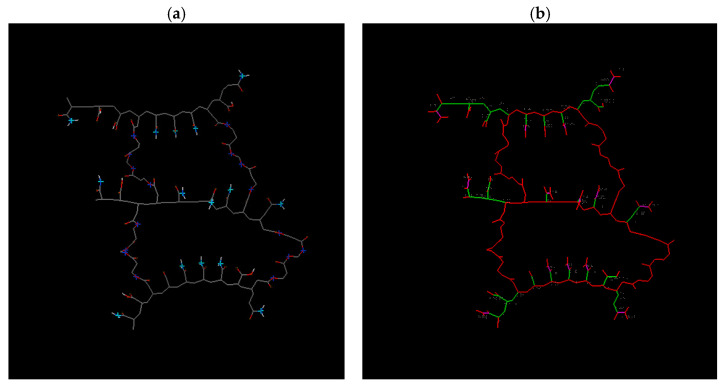
Results from Model 3 (4-HDDA model): (**a**) Hydrogen bonding donor and acceptor sites. (Light blue: acceptor, dark blue: donor, gray: carbon-carbon bond, red: oxygen atom, white: hydrogen atom). (**b**) Rotatable bonds of this model as displayed by the docking software. Green: rotatable bond, red: unrotatable bond, magenta: non-rotatable bond; 50 rotatable bonds.

Increasing the degree of crosslinking by raising the HDDA concentration results in a rise in hydrogen bonding at the donor site and a decline in the number of rotatable bonds, as illustrated in Figure 9 and Figure 10. Models 2 and 3, nonetheless, have 91 and 50 rotatable bonds, respectively. It should be noted that enhancing the crosslinking degree lowers the ES and influences water diffusion in the copolymer hydrogels. Although the crosslinking densities are not the same, dye retention is only minimally impacted. This phenomenon is the result of water diffusion within the copolymer hydrogels, with the dye being held on the surface of the copolymer hydrogel. Please refer to Table 3 for additional details.

#### 2.4.2. Hydrogen Bonding Interaction

**Figure 11:**
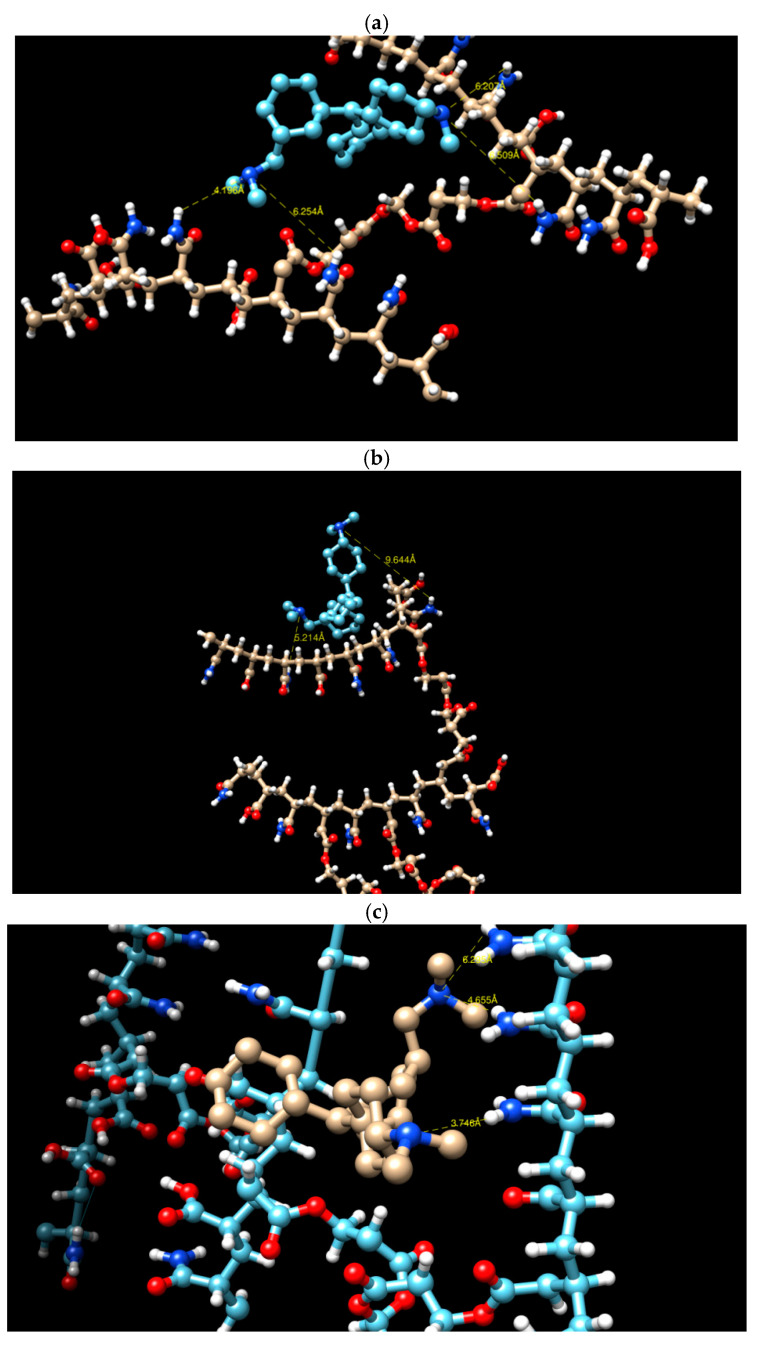
(**a**–**c**) The hydrogen bonding interaction in Models 1, 2 and 3, respectively. Blue: Nitrogen atom, white: hydrogen atom, red: oxygen atom, yellow: hydrogen bond interaction.

Figure 11 illustrates the flexibility of the molecular groups due to their possession of six rotatable bonds, facilitating their conformation and interaction with the copolymer hydrogel. Although a weak hydrogen bonding was observed between the molecular groups at interatomic distances of 6, 5, and 9 Å, a strong interaction was detected at an interatomic distance of 3.74 Å (refer to Table 4).

Table 5 presents nine modes, with the first mode representing the lowest energy state that denotes it as the most favorable mode. The negative sign indicates that the interaction between the systems occurred spontaneously, without the need for additional energy. Model 1, which has low crosslinking, has an energy of −4.9 kcal/mol. This is followed by Model 3, with high crosslinking, at −4.3 kcal/mol. Model 2, with intermediate crosslinking, is the least stable at −4.2 kcal/mol.

### 2.5. Discussion of the Results with Other Work and Effects of Other Parameters

#### 2.5.1. Removal Percentage

Hua et al. [42] used biomimetic apatite hydrogel beads inspired by the structure of petunia pollen to efficiently remove MG by adsorption. Using an ionic crosslinking approach, magnetic hydroxyapatite nanoparticles (MHNPs) were encapsulated in sodium alginate gels to fabricate hydrogel beads (MHSBs) with the structure of petunia pollen. The MG adsorption capacity of MHNPs was 208.06 mg/g, which showed a satisfactory adsorption ability.

In the study of Peighambardoust et al. [28], the carboxymethyl cellulose-based graft poly(acrylamide) hydrogel (CMC-g-P(AAm)) and its nanocomposite with montmorillonite (CMC-g-P(AAm)/MMT) were prepared by the radical method and used to remove MG dye from an aqueous solution. The maximum monolayer adsorption capacity (q_max_) determined by the Langmuir isotherm model for CMC-g-P(AAm) and CMC-g-P(AAm)/MMT were found to be 158.1 mg/g and 172.4 mg/g, respectively.

In our prior research [22], EY and RB dye molecules dissociated into negatively charged ions in an aqueous solution. The dyes possessed a robust hydrogen bond with the polyAM-co-AA/HDDA copolymer network. After 24 h, the retention rates for RB and EY were 98% and 50%, respectively. Notably, in the current study, MG demonstrated a retention rate of about 96% for a contact period of one hour. The EY and RB dyes are anionic, while MG is cationic. The ionic bonds between the molecules can significantly affect and enhance retention [43,44]. Our perspective on retention differs from the traditional viewpoint of electrostatic attraction between molecules. Most studies on the removal of MG tend to focus on retention rates by manipulating internal and external factors, rather than using simulation to estimate affinity in the polymer/dye system. However, this study delves into the nature of the interaction between dye and polymer chains, exploring new factors like rotatable bonds and donor/acceptor of hydrogen bonds.

The absorbance increased after one hour, suggesting that MG was released. An external factor seems to be accountable for this outcome. The acidic medium’s pH facilitates the release of dye. Yinuo et al. [45] successfully employed a novel pH and temperature dual-responsive hydrogel (TZP-hydrogel) to efficiently extract and release the MB dye from aqueous solutions. The MB release rate can surpass 91% at pH = 1 and 60 °C, making TZP-hydrogel reusable and recyclable. This study showcases the potential for developing recyclable hydrogel adsorbents by highlighting their unique irreversible on/off switching property. Our hydrogel boasts exceptional properties because MG is released within an hour of contact time, enabling its suitability for recycling.

The polymeric material poly(AM-co-AA)/HDDA demonstrates superior MG retention due to its benefits such as easy production, shorter contact duration, and the absence of external parameter variations that typically demand more energy and chemical products, ultimately leading to increased industrial operational costs.

#### 2.5.2. pH Effect

The environmental pH significantly impacts the swelling behavior of the poly(AM-co-AA) hydrogel. Seddiki et al. [46] discovered that the percentage of swelling changes in response to variations in the medium’s pH. The system exhibits a basic pH within the pH range of 7.0–10.0, leading to an escalation in the concentration of basic cations in the outer solution. Consequently, the mobile ion concentration increases at a faster rate in comparison to the outer solution. Thus, a significant transition was observed within this pH range. Swelling declined above pH = 7.0 due to the potential dissociation of -COOH groups and an increase in mobile ion concentration, resulting in a reduction in osmotic pressure. In an acidic medium, a marked decrease in swelling was observed. The hydrogel exhibits high swelling at a neutral pH of 7. This can be attributed to the increased diffusion of water, which in turn facilitates the retention of dye in the hydrogel.

Imran et al. [47] established that MG exhibits low solubility in extremely acidic and basic environments (pH = 2 and 12, correspondingly). The optimal solubility occurs when pH ranges from 6 to 8.

Alqadami et al. [48] investigated the applicability of JP/SB@4:1 hydrochar for the removal of MG compound and other dyes. Their findings revealed that at 298 K and in the pH range of 2.2–8.8, JP/SB@4:1 hydrochar can adsorb 25 mg/L of cationic dye solutions. The adsorption capacity of MG dye on JP/SB@4:1 increased from 69.5 to 156 mg/g as pH rose from 2.2 to 7.6. However, further increase to pH = 8.8 showed no visible increase. Changes in MG dye solution color were observed above pH = 7.6 due to a reaction between the dye and OH ions. Thermodynamic parameters demonstrate that the adsorption process is feasible, spontaneous, and endothermic. The mechanism for dye adsorption involves electrostatic interaction, hydrogen bond interaction, and π − π/n − π interaction.

In conclusion, MG has a low absorbance in both highly acidic and basic media. Nonetheless, at pH = 6 and 8, MG presents a good absorbance rate, and the hydrogel also exhibits high swelling within this range. Therefore, the selection of a neutral pH in this study could result in increased retention, possibly attributed to the favorable MG solubility and the high swelling capacity of the hydrogel.

#### 2.5.3. Water Quality Effect

Betraoui et al. [49] conducted a study on the swelling degree of AAc-graf-Agar and AAm-graf-Agar hydrogels in both deionized water and salt water containing 0.9 wt % NaCl. The AAc-graf-Agar hydrogel reached a swelling equilibrium of 6000 wt % and 2800 wt % in deionized water and salt water, respectively. On the other hand, the AAm-graf-Agar hydrogel demonstrated a swellability of 5700 wt % and 3600 wt % in deionized and salt water, respectively. Higher swelling capacity can be attributed to the presence of carboxylic acid groups (-COO) along the macromolecular chains of poly(AA) and the increase in the number of free hydrogen ions (counter ions) in the gel phase. This results in increased chain relaxation, as similarly charged -COO groups repel.

The presence of electrolyte salts impedes swelling due to ex-osmosis, and even swollen hydrogels significantly contract in the presence of salts. This phenomenon is a result of the Na^+^ counter-ion’s action causing the collapse of the polymer’s internal network. With high concentration of counter-ions, such as Na^+^ ions, in this case, they condense around the fixed -COO charges, leading to a decrease in the repulsive forces between -COO groups along the polymer segments, resulting in a reduction in the degree of swelling. The rise in salt concentration of water results in an increase in osmotic pressure that moves from the lesser concentrated medium (hydrogel) to the more concentrated medium (salted water), leading to a lower percentage of swelling.

Seddiki et al. [46] reported that poly(AM-co-AA) exhibits an equilibrium swelling of 3500% in deionized water, but only 600% in salt water. Zhenyu et al. [50] conducted a study on the distinct effects of NaCl concentration on the absorption of Dex-MA/P(AA) hydrogel. The results revealed that an increase in NaCl concentration from 0 to 400 mM caused a change in the removal efficiency of MB from >97% to 4.3%, and that of CV from >97% to 43.9%. This is in line with Na^+^ ions competitively interacting with the carboxylate groups of Dex-MA/P(AA), generating a screening effect that lowers the adsorption of cationic dyes.

Accordingly, the removal percentage of MG by the poly(AM-co-AA) will likely decrease in saltwater compared to distilled water utilized in this study.

#### 2.5.4. Temperature Effect and Recycling of the Hydrogel

Mousavi et al. [51] discovered that as the temperature increased from 298 K to 318 K, the adsorption of MB on P(AA) rose from 70 mg/g to 93 mg/g. This implies an endothermic removal process. The increase in mobility of the dye with rising temperatures may be a contributing factor. In addition, an increasing number of molecules can acquire enough energy to interact with active sites on the surface. In addition, higher temperatures can cause swelling in the internal structure of the P(AA), allowing larger dyes to penetrate more deeply.

Zhao et al. [52] discovered that the Polyacrylamide-PhyticAcid-Polydopamine (P(AM)/PA/P(DA)) hydrogel, a three-dimensional porous material, serves as a reusable adsorbent with high efficiency for both anionic and cationic dyes. The model dyes used were MB, methylene blue (YMB), methyl violet (MV), and neutral red (NR). To test its regenerative ability, adsorption and desorption experiments were conducted with the P(AM)/PA/P(DA) hydrogel using dyes. The dye can be easily desorbed by adjusting the solution pH values (i.e., pH = 2.0 for cationic dyes such as NR, MV, and YMB and pH = 11.0 for the anionic dye MB). Equilibrium absorption capacities remained stable after seven cycles of adsorption–desorption, indicating consistent dye removal and recovery. The P(AM)/PA/P(DA) hydrogel can be easily separated from water through pH value adjustments post-adsorption.

A biocompatible hydrogel composed of Dex-MA/P(AA) was synthesized by Zhenyu et al. [50] via the copolymerization of glycidyl methacrylate substituted dextran (Dex-MA) with AA. This hydrogel was utilized as an adsorbent for the elimination of cationic dyes from aqueous solutions. The MB and CV removal efficiencies achieved 93.9% and 86.4%, respectively, within one minute at an initial concentration of 50 mg/L. The elimination rates for MB and CV remained >95% even after undergoing five adsorption/desorption cycles.

The copolymer poly(AM-co-AA) investigated in this study displays the capacity to remove the cationic dye MG and can be reused over five cycles.

## 3. Conclusions

The study demonstrated significant removal of MG after 1 h when using the poly(AM-co-AA)/HDDA copolymer hydrogel. The absorbance remained constant despite variations in HDDA concentration, indicating surface retention of the dye. Swelling extent in water at equilibrium was strongly affected by crosslinking degree, with the hydrogel attaining an ES of 96% at 1 wt % HDDA. On the other hand, the percentage was 67% with 4 wt % HDDA and 48% with 7 wt % HDDA, suggesting adsorption rather than absorption of the dye and absorption of water.

The ionic interactions analysis revealed the positive charge of MG, indicating its cationic nature. The ionic copolymer hydrogel, characterized by a negative charge, attracts MG via opposite charges, leading to the creation of ionic bonds with the copolymer. The anionic nature of the carboxyl group COO^−^ in the repeating unit of AA attracts the opposite charge of MG, resulting in the creation of coulombic forces between opposite charges. The high number of rotatable bonds in this dye, along with its two hydrogen bonding donor sites, increases its retention.

The copolymer hydrogels’ high retention of MG was investigated through a simulation. Three different crosslinking models were analyzed, revealing a decreasing number of rotatable bonds with higher amounts of HDDA. MG is characterized by two hydrogen bonding sites that serve as donors, whereas the copolymer model contains a significant number of acceptor sites for hydrogen bonding. The retention of MG within the polymeric substance is responsible for this effect.

The crosslinked poly(AM-co-AA) using HDDA shows promise as an effective method for removing MG from aqueous solutions, achieving a 96.4% removal rate within an hour at room temperature. The copolymer acts as an HBA, while the MG functions as an HBD. With 121 rotatable bonds in the copolymer and six in the dye, there is flexibility in the dye/copolymer configuration, facilitating improvements in the adsorption process.

## 4. Materials and Methods

### 4.1. Materials

The research employed two monomers, identified as AM and AA, both obtained from Sigma-Aldrich, Saint-Quentin-Fallavier, France, and possessing a purity level of 99%. The crosslinking agent utilized was HDDA from Cray Valley located in Courbevoie, France, with a 98% purity level. The photoinitiator used was 2-hydroxy-2-methyl-1-phenyl-propane-1 (commercially known as Darocur 1173) sourced from Ciba-Geigy, Basel, Switzerland, with a purity of 97%. Additionally, MG dye obtained from Sigma-Aldrich with a purity level of 70% was implemented. All the products were utilized without the need for their purification.

### 4.2. Preparation of Photochemically Crosslinked Copolymers

The AM and AA were stirred together in equal amounts (at a 1:1 mass ratio) in distilled water (pH = 6.7) at room temperature (T = 24 °C) for 24 h to prepare the AM/AA solution. The resulting solution was then supplemented with the crosslinker HDDA at differing concentrations (1 wt %, 4 wt %, and 7 wt %), as detailed in Tables S5 and S6. Furthermore, Darocur 1173 was included as a photoinitiator at 0.5 wt %, as presented in Table 6. The solutions were placed in a Teflon mold and exposed to UV radiation for 30 min, utilizing a Philips TL08 blacklight with two 18 W bulbs producing maximum emission at 365 nm. Nitrogen was circulated to evacuate the air atmosphere from the Teflon mold during the process of polymerization-crosslinking. The outcome yielded transparent pellets, exemplifying three-dimensional cross-linked polymer networks. Please refer to Table 6 for all formulations.

A quantity of 1.3 mg of MG was measured for each experiment and added to a beaker containing 450 mL of distilled water. The solution was stirred for 24 h.

### 4.3. Study of the Swelling of the Copolymer Hydrogels in the MG Solution

The analysis of swelling in the copolymer hydrogels was conducted using distilled water with MG. Equation (2) [53,54] was utilized to compute the parameter *ES*, which represents the equilibrium swelling state after prolonged swelling periods.

(2) $$ES=\left(\frac{{m_{t}-m}_{0}}{m_{0}}\right)100$$

The pellet’s mass in the swollen state at time t is denoted as m_t_, while m_0_ represents the initial mass of the pellet in the dry state at t = 0. The process entailed weighing three pellets of the copolymer networks initially with differing percentages (1 wt %, 4 wt %, and 7 wt %) of HDDA. The pellets were then immersed into three beakers, each filled with 150 mL of excess solvent with the dye solution. This was performed within the laboratory setup at 24 °C. The experiment commenced by employing a stopwatch to measure the kinetics of swelling in the crosslinked hydrogels on an hourly basis.

After a defined immersion period, three enlarged pellets were extracted from the beaker and precisely weighed on a scale with an accuracy of ±0.001 g. Simultaneously, solutions were periodically examined using a UV-visible spectrophotometer (Specord 200 plus, Analytik Jena, Jena, Germany) in order to evaluate their absorbance. The samples were then returned to the beaker and the swelling behavior was regularly observed until the copolymeric hydrogels reached saturation.

### 4.4. Model Proposition and Software

Three models were analyzed, each consisting of three chains of copolymers composed of both AM and AA monomers. Each chain had 5 AM monomers and 5 AA monomers. Model 1 contained a copolymer of this type with two difunctional HDDA monomers. The final model underwent geometric minimization via the Avogadro software, yielding a minimum energy of E = 87,928.5 kJ/mol and a difference in energy of dE = 67.6 kJ/mol. Model 2 and Model 3 were created utilizing the identical composition as Model 1, with the only variation being the inclusion of three and four HDDA molecules in each, respectively. Model 2 had a minimum energy of E = 38,351.3 kJ/mol and an energy difference of dE = 172.5 kJ/mol, while Model 3 had a minimum energy of E = 50,330.3 kJ/mol and a dE of 85.2 kJ/mol. The software and docking methodology utilized in this study are explained thoroughly in reference [21].

## Figures and Tables

**Table 1:** Ranking of poly(AM-co-AA) hydrogel with varying AM and HDDA concentrations for descending MG removal.

Copolymer Network	MG Removal (%)
Poly(AM_C2_-co-AA)/4 wt % HDDA	96.4
Poly(AM_C2_-co-AA)/7 wt % HDDA	95.7
Poly(AM_C2_-co-AA)/1 wt % HDDA	95.5
Poly(AM_C1_-co-AA)/4 wt % HDDA	93.1
Poly(AM_C1_-co-AA)/7 wt % HDDA	91.0
Poly(AM_C1_-co-AA)/1 wt % HDDA	89.0
Poly(AM_C3_-co-AA)/7 wt % HDDA	85.3
Poly(AM_C3_-co-AA)/1 wt % HDDA	84.5
Poly(AM_C3_-co-AA)/4 wt % HDDA	83.7

**Table 2:** Equilibrium swelling (ES) after 24 h of immersion of poly(AM-co-AA) hydrogel in MG solution for different initial concentrations of AM and HDDA.

	Equilibrium Swelling (ES) (%)		
	Poly(AM_C1_-co-AA)	Poly(AM_C2_-co-AA)	Poly(AM_C3_-co-AA)
1 wt % HDDA	96.5	89.1	97.9
4 wt % HDDA	72.7	40.6	72.9
7 wt % HDDA	67.6	20.1	51.9

**Table 3:** Donor and acceptor sites, and rotatable bonds for each model and MG.

Models	Hydrogen Bond Acceptor (HBA)	Hydrogen Bond Donor (HBD)	Rotatable Bond
Model 1	16	7	121
Model 2	16	12	91
Model 3	16	16	50
MG	0	2	6

**Table 4:** Hydrogen bonding interaction in the three models.

Models	Hydrogen Bond	Distance (Å)
Model 1	N……H	4.19
		6.25
		6.50
		6.20
Model 2	N……H	5.21
		9.64
Model 3	N……H	3.74
		4.65
		6.29

**Table 5:** Affinity of the MG/copolymer system for the nine cases of the MG conformation. Rmsd l. b. stands for «Root mean square deviation lower bound», and is often used to measure the quality of the reproduction of a binding pose by a computational method, such as docking.

Models	Mode	Affinity (kcal/mol)	Dist. from Rmsd l.b. (Å)
Model 1			
	1	−4.9	0.000
	2	−3.7	14.657
	3	−3.5	14.084
	4	−3.5	13.731
	5	−3.4	6.865
	6	−3.3	6.035
	7	−3.3	9.936
	8	−3.3	2.641
	9	−3.2	12.364
Model 2			
	1	−4.2	0.000
	2	−4.1	14.630
	3	−4.1	21.335
	4	−3.3	18.793
	5	−3.2	5.862
	6	−3.2	35.362
	7	−3.2	7.697
	8	−2.9	33.130
	9	−2.7	31.404
Model 3			
	1	−4.3	0.000
	2	−3.7	5.015
	3	−3.4	12.720
	4	−3.3	22.798
	5	−3.2	14.812
	6	−2.9	8.349
	7	−2.9	15.384
	8	−2.8	17.327
	9	−2.7	22.691

**Table 6:** Formulation of the elaborated crosslinked copolymers.

Copolymer Network *	Solution AM (mg/mL)	Darocur 1173 (wt %)	HDDA (wt %)
PAM_C1_-co-AA	134	0.5	1
			4
			7
PAM_C2_-co-AA	100	0.5	1
			4
			7
PAM_C3_-co-AA	67	0.5	1
			4
			7

* The amount of acrylic acid remains constant at a 50:50 weight ratio for AM:AA mixtures.

## Data Availability

The data set presented in this study is available in this article.

## Supplementary Materials

The following supporting information can be downloaded at: https://www.mdpi.com/article/10.3390/gels9120946/s1, Figure S1. Absorbance of different MG concentrations at room temperature (T = 24 °C) in aqueous medium (distilled water, pH = 6.7); Figure S2. Calibration curve of MG (see Table 1); Table S1. Concentration of MG versus the absorbance at 617 nm; Table S2. Absorbance as a function of contact time applying C_1_ as initial concentration of AM; Table S3. Absorbance as a function of contact time applying C_2_ as initial concentration of AM; Table S4. Absorbance as a function of contact time applying C_3_ as initial concentration of AM; Table S5. Preparation of the initial AM solutions; Table S6. Quantities of (AM/AA) solution, HDDA and Darocur 1173.
