# Supplementary material for: Removal of Malachite Green by Poly(acrylamide-co-acrylic acid) Hydrogels: Analysis of Coulombic and Hydrogen Bond Donor–Acceptor Interactions
Source: Gels. 2023 Dec 1;9(12):946. doi: 10.3390/gels9120946 (PMC10742954; doi:10.3390/gels9120946)
Supplement: Supplementary file 1 [file gels-09-00946-s001.zip › gels-2688763-supplementary.pdf]

SUPPLEMENTARY MATERIAL

Removal of Malachite Green by Poly(acrylamide-co-acrylic acid) Hydrogels : Analysis of Coulombic and Hydrogen Bond Donor-Acceptor Interactions

Salah Hamri [1,2], Bouchra Bouzi [2], Djahida Lerari [1], Fayçal Dergal [1], Tewfik Bouchaour [2], Khaldoun Bachari [1], Zohra Bouberka [3] and Ulrich Maschke [4,*]

[1] Center for Scientific and Technical Research in Physico-Chemical Analysis (CRAPC), BP 384, Industrial Zone, 42004 BouIsmaïl, Algeria
[2] Macromolecular Research Laboratory (LRM), Faculty of Sciences, Abou Bekr Belkaid University, BP 119, 13000 Tlemcen, Algeria
[3] Laboratoire Physico-Chimie des Matériaux-Catalyse et Environnement (LPCMCE), Université des Sciences et de la Technologie d'Oran Mohamed Boudiaf (USTOMB), BP 1505, 31000 Oran, Algeria
[4] Unité Matériaux et Transformations – UMET, UMR 8207, Université de Lille, CNRS, INRAE, Centrale Lille, 59000 Lille, France
* Correspondence: ulrich.maschke@univ-lille.fr

SUPPLEMENTARY MATERIAL

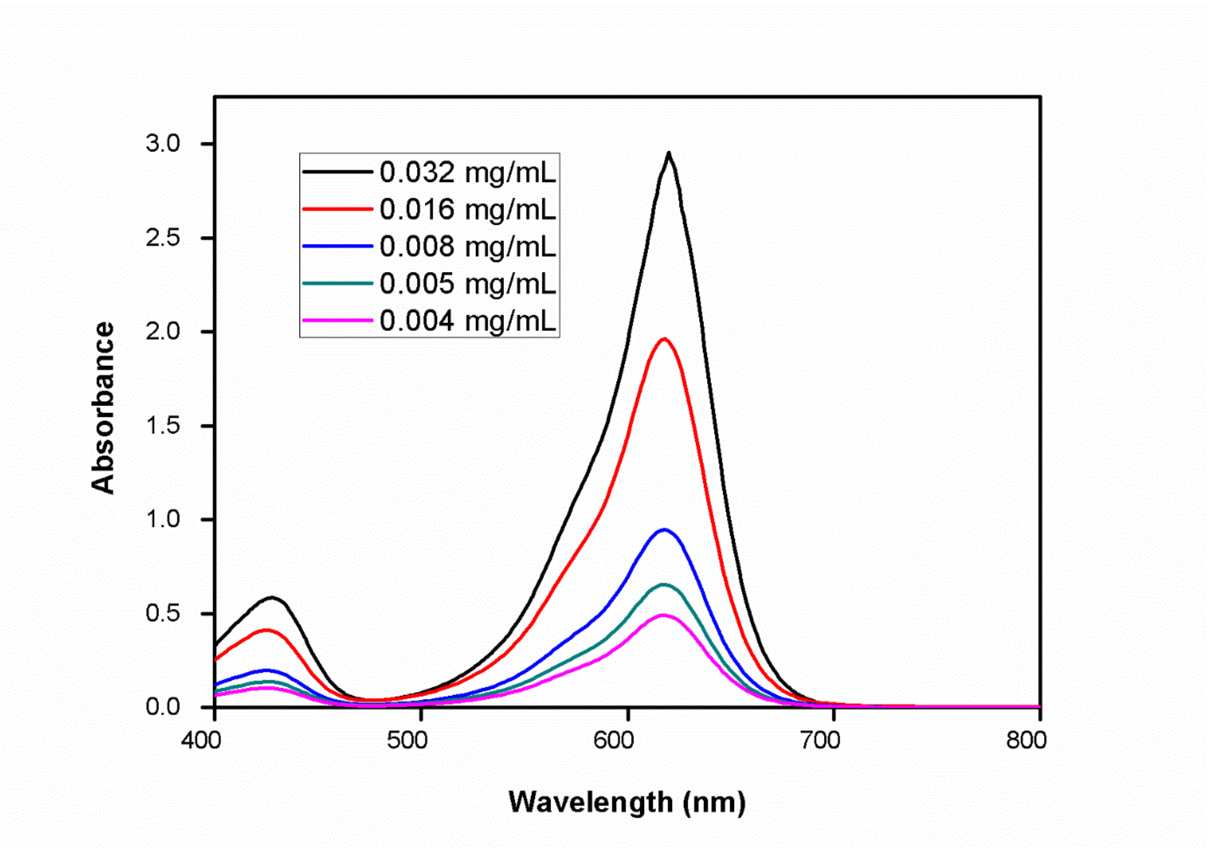

Figure S1. Absorbance of different MG concentrations at room temperature (T=24°C) in aqueous medium (distilled water, pH=6.7).

SUPPLEMENTARY

Table S1. Concentration of MG versus the absorbance at 617nm.

Concentration mg/mL	0.032	0.016	0.008	0.00533	0.004
Absorbance	2.95	1.96	0.94	0.65	0.49

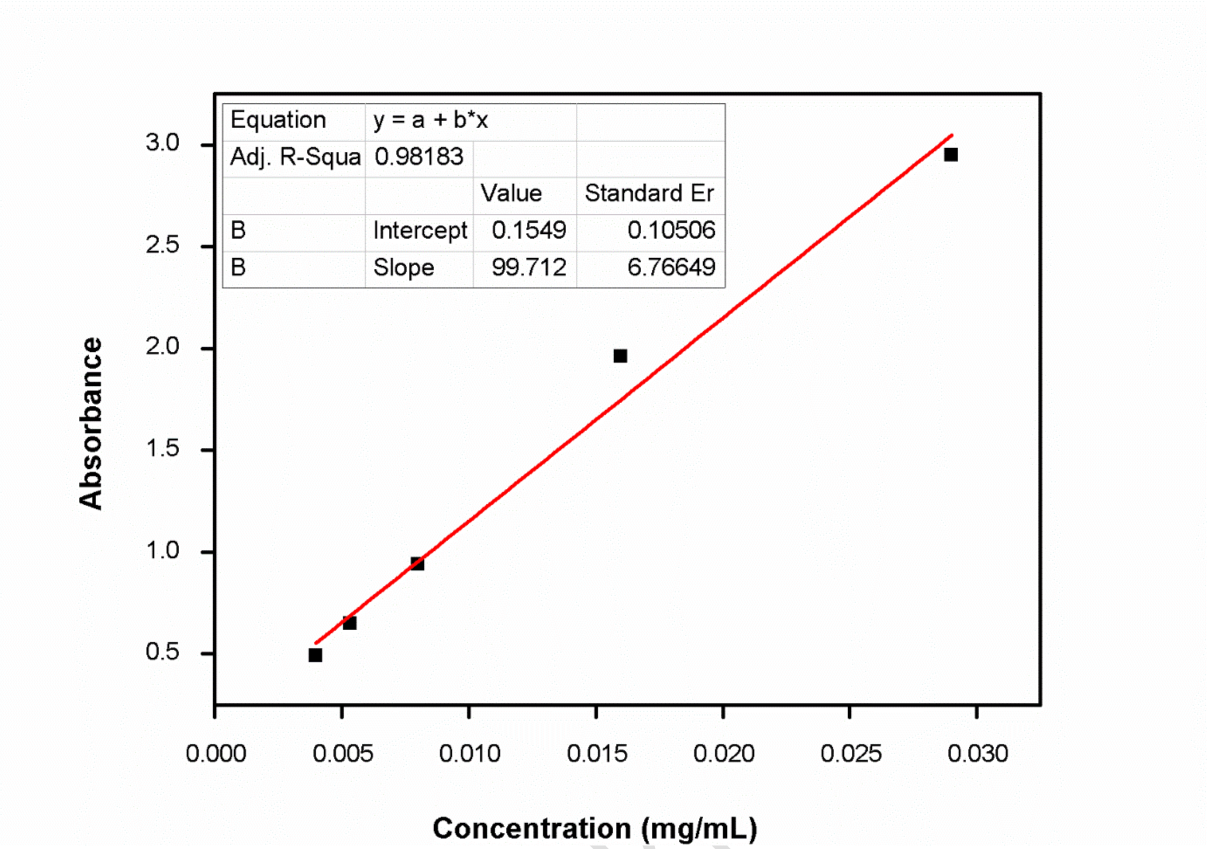

Figure S2. Calibration curve of MG (see table 1).

SUPPLEMENTARY

Table S2. Absorbance as a function of contact time applying C_1 as initial concentration of AM.

Contact Time (h)	0	1	2	3	4	24
Abs 1 wt% HDDA	2.76	0.30	0.42	0.40	0.29	0.29
Abs 4 wt% HDDA	2.76	0.19	0.37	0.37	0.27	0.28
Abs 7 wt% HDDA	2.76	0.25	0.39	0.41	0.28	0.28

Table S3. Absorbance as a function of contact time applying C_2 as initial concentration of AM.

Contact Time (h)	0	1	2	3	4	24
Abs 1 wt% HDDA	2.95	0.12	0.25	0.17	0.25	0.29
Abs 4 wt% HDDA	2.95	0.09	0.15	0.17	0.21	0.28
Abs 7 wt% HDDA	2.95	0.12	0.16	0.19	0.20	0.27

Table S4. Absorbance as a function of contact time applying C_3 as initial concentration of AM.

Contact Time (h)	0	1	2	3	4	24
Abs 1 wt% HDDA	2.80	0.43	0.44	0.43	0.42	0.20
Abs 4 wt% HDDA	2.80	0.45	0.40	0.40	0.39	0.20
Abs 7 wt% HDDA	2.80	0.41	0.40	0.40	0.38	0.25

Table S5. Preparation of the initial AM solutions.

Solution (AM)	01	02	03
Mass of Acrylamide (g)	20	15	10
Volume of distilled water (mL)	150	150	150
Concentration (mg/mL)	133.33	100	66.66
	C_1	C_2	C_3

Table S6. Quantities of (AM/AA) solution, HDDA and Darocur 1173.

Solutions	Solution 01	Solution 02	Solution 03
AM/AA sol.	98.5%	95.5%	92.5%
HDDA	1%	4%	7%
Darocur 1173	0.5%	0.5%	0.5%

SUPPLEMENTARY MATERIAL
